# Protein Phosphatase 2B Dual Function Facilitates Synaptic Integrity and Motor Learning

**DOI:** 10.1523/JNEUROSCI.1741-20.2021

**Published:** 2021-06-30

**Authors:** Zhanmin Lin, Bin Wu, Maarten W. Paul, Ka Wan Li, Yao Yao, Ihor Smal, Martina Proietti Onori, Hana Hasanbegovic, Karel Bezstarosti, Jeroen Demmers, Adriaan B. Houtsmuller, Erik Meijering, Freek E. Hoebeek, Martijn Schonewille, August B. Smit, Zhenyu Gao, Chris I. De Zeeuw

**Affiliations:** ^1^Department of Neuroscience, Erasmus MC, 3015 GE, Rotterdam, The Netherlands; ^2^Department of Neurology and Institute of Neurology, Huashan Hospital, Fudan University, 200040, Shanghai, China; ^3^Optical Imaging Center, Erasmus MC, 3015 GE, Rotterdam, The Netherlands; ^4^Department of Molecular and Cellular Neurobiology, Center for Neurogenomics and Cognitive Research, VU University Amsterdam, Amsterdam Neuroscience, 1081 HV, Amsterdam, The Netherlands; ^5^Department of Medical informatics, Erasmus MC, 3015 GE, Rotterdam, The Netherlands; ^6^Center for Proteomics, Erasmus MC, 3015 GE, Rotterdam, The Netherlands; ^7^School of Computer Science and Engineering & Graduate School of Biomedical Engineering, University of New South Wales, Sydney, 2052, New South Wales, Australia; ^8^Department for Developmental Origins of Disease, Wilhelmina Children's Hospital and Brain Center, Utrecht Medical Center, 3584 EA, Utrecht, The Netherlands; ^9^Netherlands Institute for Neuroscience, KNAW, 1105 BA, Amsterdam, The Netherlands

**Keywords:** cerebellar learning, protein phosphatase 2B, Purkinje cells

## Abstract

Protein phosphatase 2B (PP2B) is critical for synaptic plasticity and learning, but the molecular mechanisms involved remain unclear. Here we identified different types of proteins that interact with PP2B, including various structural proteins of the postsynaptic densities (PSDs) of Purkinje cells (PCs) in mice. Deleting PP2B reduced expression of PSD proteins and the relative thickness of PSD at the parallel fiber to PC synapses, whereas reexpression of inactive PP2B partly restored the impaired distribution of nanoclusters of PSD proteins, together indicating a structural role of PP2B. In contrast, lateral mobility of surface glutamate receptors solely depended on PP2B phosphatase activity. Finally, the level of motor learning covaried with both the enzymatic and nonenzymatic functions of PP2B. Thus, PP2B controls synaptic function and learning both through its action as a phosphatase and as a structural protein that facilitates synapse integrity.

**SIGNIFICANCE STATEMENT** Phosphatases are generally considered to serve their critical role in learning and memory through their enzymatic operations. Here, we show that protein phosphatase 2B (PP2B) interacts with structural proteins at the synapses of cerebellar Purkinje cells. Differentially manipulating the enzymatic and structural domains of PP2B leads to different phenotypes in cerebellar learning. We propose that PP2B is crucial for cerebellar learning via two complementary actions, an enzymatic and a structural operation.

## Introduction

The maintenance and modulation of synaptic transmission are critical for virtually all brain functions, varying from online control of perception and action up to long-term processes, such as memory formation and retrieval. One of the main cellular mechanisms by which neurons control synaptic efficacy is to dynamically set the phosphorylation state of synaptic proteins (Feng and Zhang, 2009). Phosphorylation is achieved by activation of protein kinases, such as PKA, PKC, and CaMKII (Hell, 2014), whereas dephosphorylation is accomplished by protein phosphatases (Shi, 2009). Phosphoprotein phosphatase 2B (PP2B, calcineurin), which predominantly dephosphorylates proteins on Ser and Thr residues, accounts for up to 1% of the total protein in mammalian neurons and is enriched in synapses (Stemmer and Klee, 1991; Kuno et al., 1992).

PP2B phosphatase activity is triggered by binding of the Ca^2+^/calmodulin complex (Mansuy and Shenolikar, 2006; Shi, 2009), which leads to dephosphorylation of synaptic and cytoplasmic proteins in a Ca^2+^-dependent manner. Depending on synaptic Ca^2+^ influx and the resulting Ca^2+^ levels, PP2B is activated and thought to functionally counteract protein kinases PKA and CaMKII, which together provide a complex phosphorylation/dephosphorylation signature of proteins (Stemmer and Klee, 1991). Thereby, the Ca^2+^ concentration can determine the state of phosphorylation and dephosphorylation of synaptic proteins and change their functional status. For example, the precise phosphorylation state of AMPARs determines to what extent they will be subject to exocytosis or endocytosis (Jörntell and Hansel, 2006). Such a Ca^2+^-dependent dynamic switch of the phosphorylation state of AMPARs has been postulated to determine the threshold and direction for the induction of long-term plasticity, that is, controlling the level of LTP or LTD (also referred to as the Bienenstock-Cooper-Munro plasticity rule) (Bienenstock et al., 1982), which has been validated in various brain regions. In hippocampal pyramidal cells, PP2B directly dephosphorylates Ser845 of the AMPA-type GluR1 subunit (Jurado et al., 2010; Sanderson et al., 2012), which in turn regulates receptor insertion and removal from the membrane, underlying induction of LTP and LTD, respectively (Roche et al., 1996). Likewise, for cerebellar Purkinje cells (PCs), synaptic plasticity has also been shown to depend on proper functioning of PP2B (Malleret et al., 2001; Zeng et al., 2001; Mansuy, 2003).

Interestingly, over the past decade CaMKII has been shown to operate not only as an enzyme (i.e., a kinase) but also as a structural synaptic protein (Shen et al., 1998; Hell, 2014). For example, the presence of kinase-dead CaMKII is sufficient for normal short-term presynaptic plasticity and activity-dependent proteasome redistribution in hippocampal neurons, indicating its kinase activity is not essential for these functions (Hojjati et al., 2007; Bingol et al., 2010). Given the enrichment of PP2B in the synapse (Kuno et al., 1992) and its counteracting function to CaMKII, we set out to test the hypothesis that PP2B may control, like CaMKII, synaptic functions not only via its enzymatic activity, but also through a mechanism independent thereof. Here we show that blocking the enzymatic function of PP2B only partially affects cerebellar motor learning, that is, adaptation of the vestibulo-ocular reflex (VOR), whereas full genetic ablation of native PP2B from PCs (*L7-PP2B* KO mice) completely annihilates this. In addition, PP2B in PCs, but not in granule cells (GCs), interact with proteins such as Shank2, Homer3, and mGluR1, all of which are prominently localized at or near the postsynaptic density (PSD) of their parallel fiber (PF) inputs. These proteins remain properly localized at the PF to PC synapse as long as PP2B is present, also in its enzyme-deficient form. Accordingly, when we genetically ablate PP2B from PCs, the ultrastructure of the PSDs of their PF inputs is affected, showing a reduction of their thickness relative to their length. On the other hand, the lateral mobility of AMPARs across the surface of synapses of PC spines appeared to depend entirely on the phosphatase activity of PP2B. The combined enzymatic and nonenzymatic actions of PP2B were also revealed when we bilaterally infected the flocculi of the vestibulocerebellum of *L7-PP2B* KO mice with enzyme-dead PP2B in that this approach partially rescued VOR learning. Together, our data show that PP2B does not only operate as an enzyme, but also serves a critical role in controlling PSD protein levels and structure, highlighting a dual function of PP2B in governing synaptic plasticity and learning.

## Materials and Methods

### 

#### 

##### Animals

We focused on the use of *L7-PP2B* KO mice, in which PP2B is specifically knocked out in PCs (Schonewille et al., 2010). In addition, we generated a granule cell -specific PP2B KO by crossing the *floxed-PP2B* mice with α*6-Cre* mice, and we generated a global, inducible PP2B KO for control by crossing the *floxed-PP2B* mice with *Cre^ER2^* mice. Unless stated otherwise, male mice of the following genotypes were used for the experiments: homozygous/+ (referred to as L7-PP2B or α*6*-PP2B) and homozygous/–, WT/+ and WT/– (littermate controls). All mice were of the *C57/Bl6* background.

##### Statistics

**Figure 1.**
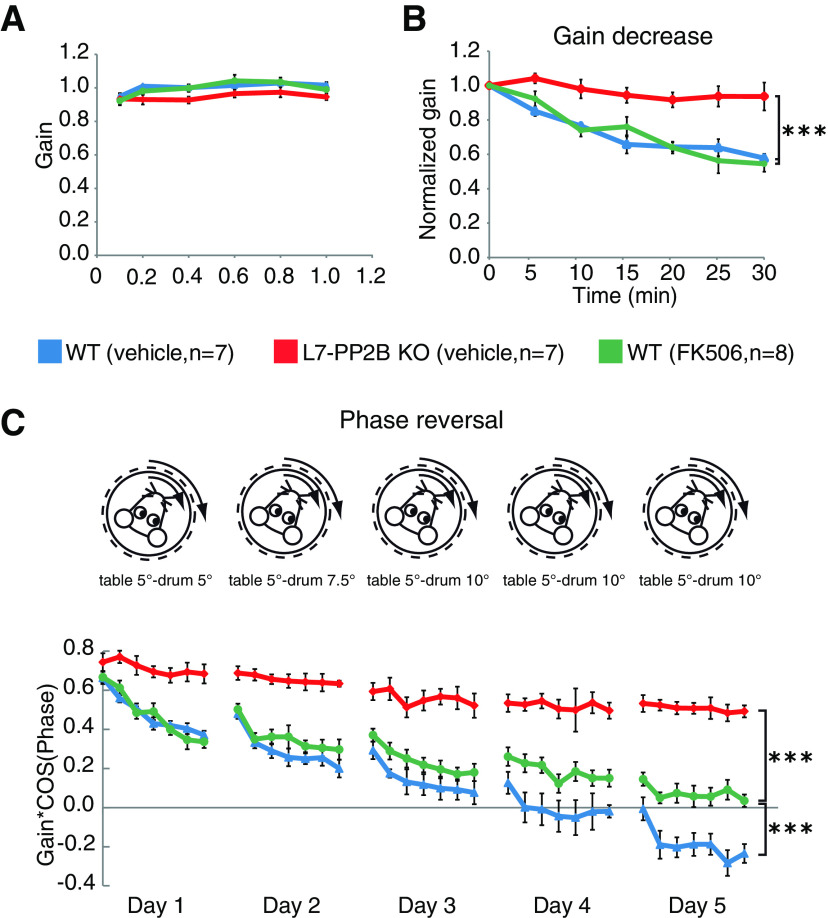
Selective inhibition of phosphatase activity of PP2B does not fully reproduce the phenotype of the *L7-PP2B KO*. ***A***, Motor performance during the VOR in the light (VVOR) revealed no aberrations in the FK506-injected group (i.e., group in which phosphatase function of PP2B was specifically inhibited) compared with controls and *L7-PP2B KO*s (all *p* values > 0.5). Error bars indicate ± SEM. ***B***, Short-term learning paradigm of gain decrease. *L7-PP2B KO* mice were unable to learn (***p values < 0.001 with respect to both vehicle-only and FK506 group). Error bars indicate ± SEM. ***C***, Long-term learning paradigm of 5 d phase-reversal eye movement. *y* axis indicates the value of gain*cos(phase); the lower this value, the better the learning. Phase-reversal learning in the FK506-injected group is impaired compared with controls (*p* < 0.001) but is better than that of *L7-PP2B KO* mice (*p* < 0.001), suggesting that, in addition to its function as a phosphatase, PP2B may also have a structural role. Error bars indicate ± SEM.

All statistical tests are defined in the relevant figure legends. Two-tailed paired or unpaired Student's *t* tests, unless stated otherwise. Two-way ANOVA with repeated measures was used in Figure 1 and Figure 7 for eye movement. Numerical values are provided as the mean ± SEM, and differences were considered statistically significant at *p* < 0.05, unless stated otherwise.

##### Eye movement recordings

Mice were surgically prepared under general anesthesia with isoflurane/O_2_. A construct with two nuts was attached to the frontal and parietal bones using Optibond (Kerr) and Charisma (Heraeus Kulzer). After a minimum of 3 d of recovery, mice were placed in a restrainer with the head construct fixed to a metal bar. The restrainer was fixed onto a turntable (diameter 60 cm), surrounded by a cylindrical screen (diameter 63 cm) with a random-dotted pattern. Before experiments, the animals received one training session (1 h in the restrainer) to habituate to the experimental settings. Optokinetic reflex and vestibulo-ocular reflex (VOR, in light and dark) were elicited by rotating the screen and turntable, respectively, at different frequencies (AC servo-motors, harmonic drive AG). The position of table and drum were recorded by potentiometers, and the signal was digitized (CED) and stored for offline analysis. Eye movements were recorded using an infrared CCD camera fixed to the turntable (240 Hz, Iscan). The eye movements were calibrated, and the phase reversal learning was induced as previously described (Schonewille et al., 2010). Likewise, the data were analyzed as described previously (Schonewille et al., 2010; Galliano et al., 2013).

##### Antibodies and peptides

The detailed information of antibodies used in this study is summarized in Extended Fig. 1-1. The following primary antibodies and blocking peptide were used in the immunoprecipitation (IP) experiments: rabbit anti-PP2B (4 times independent experiment replicates, polyclonal, raised against the peptide with residues 450-500 of the human PP2BA protein, Genscript USA), the latter of which was also used as IP blocking peptide. Mouse anti-PP2B (3 times independent experiment replicates, monoclonal raised against amino acids 312-521 of the PP2B catalytic Aα subunit, Santa Cruz Biotechnology). The controls were IPs without antibody, with beads, which were run along the experimental groups. All antibodies used in this study are listed in the Extended Fig. 1-1.

##### Preparation of P2+ microsome fraction

For each IP, four cerebella were homogenized in a 15 ml glass potter containing ice-cold homogenization buffer (0.32 m sucrose, 5 mm HEPES, EDTA-free protease inhibitor from Roche Applied Science, pH 7.4) at 900 RPM with 12 up-and-down strokes of the piston. The lysate was centrifuged at 1000 × *g*, 4°C, for 10 min to remove the cell debris and nuclei. The supernatant was centrifuged at 100,000 × *g*, 4°C for 120 min. After ultracentrifugation, the pellet and microsomes (P2 + M) were resuspended with 25 mm HEPES buffer (pH 7.4, protease inhibitor). The protein concentration was determined by Bradford measurement (Bio-Rad) and adjusted to 10 μg/μl.

##### Extraction of protein complexes

To solubilize proteins and protein complexes, 5 mg P2 + M was mixed with a sample volume of extraction buffer (25 mm HEPES, 150 mm NaCl, 2% DDM, pH 7.4) and incubated on a rotator at 4°C for 60 min, then centrifuged at 20,000 × *g*, 4°C for 20 min. The supernatant was collected, and the pellet was resuspended in the extraction buffer (25 mm HEPES, 150 mm NaCl, 1% DDM, pH 7.4) and subsequently incubated and centrifuged as before. After that, the supernatant was pooled with the previously collected supernatant and centrifuged at 20,000 × *g*, 4°C for 20 min. The supernatant containing the extracted proteins and protein complexes was used for further IP experiments.

##### Blue native PAGE

Blue native PAGE was performed using the Novex NativePAGE Bis-Tris Gel System (Invitrogen) according to the manufacturer's protocol. Briefly, 75 μg DDM extracted sample diluted to 100 μl was centrifuged at 134 000 × *g*, 4°C for 30 min. 14 μl of the supernatant was mixed with 2 μl BN sample buffer, 10 μl of which was used to make a mixture with 0.5 μl 5% G-250, which in turn was centrifuged at 20 000 × *g*, 4°C for 20 min. 20 μl supernatant was loaded on a 3-12% Bis-Tris gel for the first dimension, non-denaturing PAGE. After running, the gel was cut and subjected to the second dimension of SDS-PAGE and analyzed with the PP2B antibody (1:1000, Genscript). The MS/MS spectra obtained from each gel slice were searched against the mouse database with MaxQuant version 1.5.2.8. To accurately quantify and determine protein distribution across the BN-gel, peptides were manually curated using Skyline. For peak picking, a selection of the most abundant and unique peptides for the proteins of interest was made. Next, the m/z and retention time at which a given peptide was identified by MaxQuant were used to select the correct MS1 peak area. This was done for each slice separately, using the same precursor, m/z, and retention time. Peptide abundance was summed per protein per slice and divided by the maximum intensity value observed for the protein across the gel. These data are shown in a heatmap as relative abundance.

##### Immunoprecipitation (IP)

We conducted the IP experiments under two conditions, including (1) IP experiments using an anti-PP2B antibody, and (2) control experiments using empty beads without primary antibodies. After extraction of the protein complexes, 10 μg antibody was added and incubated overnight on a rotator at 4°C; 40 μl Protein A&G agarose beads (Santa Cruz Biotechnology) were washed 4 times with wash buffer (25 mm HEPES, 150 mm NaCl, 0.1% DDM, pH 7.4) and added to the sample. As a control, the extract was incubated with the beads without antibody. After 1 h incubation on a rotator at 4°C, the sample was washed for 4 times with the wash buffer. SDS-PAGE loading buffer was added to the sample and boiled at 95°C for 3 min; 5 μl 30% acrylamide per sample was added to each sample to facilitate the identification of cysteine-containing peptides. The proteins were separated on a 10% gel by an SDS-PAGE system (Bio-Rad) followed by in-gel digestion. After SDS-PAGE, the gel was fixed in a sufficient amount of fixation buffer (50% ethanol, 3% phosphoric acid) for at least 1 h and washed with Milli-Q water for 4 × 15 min; and stained with Coomassie Blue G-250 for 2 h and washed with water for 4 × 15 min. The stacking gel was removed, and the separation gel was cut into 5 pieces according to the protein marker (30, 45, 60, 120 kDa) to reduce sample complexity and to facilitate the subsequent MS analysis. The gel pieces were separately transferred into a 1.5 ml Eppendorf tube and destained. The destaining was performed by adding 500 μl of 50 mm NH_4_HCO_3_/50% acetonitrile (ACN) to the gel fragments and vortexing for 20 min. The solution was removed and discarded; 500 μl 100% ACN was added and vortexed for 20 min. The solution was removed and discarded; 200 μl 50 mm NH_4_HCO_3_ was added and incubated for 5 min at room temperature; 200 μl of ACN was added and the sample was incubated overnight allowing complete destaining. The solution was discarded, and 500 μl 100% ACN was added and vortexed for 20 min, after which the solution was discarded and the gel fragments were dried in a SpeedVac for 30 min.

##### In-gel digestion

The in-gel digestion was performed by adding 160-180 μl trypsin solution (6.7 µg/ml, sequence grade; Promega) containing 50 mm NH_4_HCO_3_ and incubation at 37°C overnight. After digestion, the solution containing the tryptic peptides was collected and the remaining peptides in the tube were extracted by adding and removing 50 mm NH_4_HCO_3_/50% ACN twice. This solution was pooled and dried in the SpeedVac and stored at −20°C. Immediately before LC-MS/MS analysis, the sample was redissolved in 15 μl 0.1 m HAc and centrifuged at 20,000 × *g* for 15 min. The supernatant-containing peptides were transferred to an MS tube and analyzed with LC-MS/MS.

##### LC-MS/MS analysis

The peptides were injected into the loop of an Eksogent nano LC-ultra 1D plus HPLC system equipped with a C18 column (200 mm home-made Altima C18 analytical column, 100 μm ID 3 μm particle size). Peptides were separated using a linear gradient of 5% solvent A (0.1% acidic acid, 5% ACN) and 45% solvent B (0.1% acidic acid, 80% ACN) in 50 min. The LC system was directly coupled inline with an LTQ-Orbitrap Velos instrument (Thermo Fisher Scientific). The LTQ-Orbitrap was set to data-dependent mode to switch automatically between MS and MS/MS. MS spectra range from 330 until 2000 m/z can be acquired in the Orbitrap at an FWHM resolution of 30,000 after accumulation to 500,000 in the linear ion trap with one microscan. The five most abundant precursor ions were selected for fragmentation by CID with an isolation width of 2 DA. CID was performed in the linear ion trap after accumulation to 50,000 with 1 microscan.

##### Protein identification

MS/MS spectra were searched against a mouse database (IPI; version 3.79) with ProteinPilot software (version 3.0; Applied Biosystems; MDS Sciex) using the Paragon algorithm (version 3.0.0.0) (Shilov et al., 2007) as the search engine. The search parameters were set to cysteine modification by acrylamide and digest done with trypsin. The detected protein threshold (unused protscore; confidence) in the software was set to 0.05 to achieve 10% confidence, and identified proteins were grouped to minimize redundancy. The immunoprecipitated protein complex was analyzed with LC-MS/MS analysis, which generated a large list of proteins. The MS/MS raw data were analyzed by ProteinPilot to translate MS2 spectra into peptides (sequence). Tryptic peptides shared by multiple proteins were assigned to the winner protein. Only proteins of which unique peptides were found were taken along. Proteins not meeting these requirements were excluded from further analysis. From the IP data, IgG, trypsin, and keratin protein clusters were excluded since these are obviously contaminants of the preparation. The proteins identified as “true interactors” meet the criteria in that they needed to be identified at least twice with each antibody. Protein datasets were loaded into ingenuity pathway analysis, which was used to create a protein interaction network. All interconnections made by ingenuity pathway analysis were based on “known direct interactions.”

##### iTRAQ: synaptic membrane preparation

Synaptic membranes from predominantly glutamatergic synapses were isolated from 10 to 12 week α*6-PP2B, L7-PP2B* and WT mice as described previously (Li, 2003; Klychnikov et al., 2010). A short overview of the experimental setup is presented in Figure 3*A*. In brief, for each sample, cerebellum from 1 mouse was homogenized as described in Preparation of P2^+^ microsome fraction. The lysate was centrifuged at 1000 × *g,* 4°C, for 10 min. The supernatant was loaded on top of a sucrose step gradient consisting of 0.85 and 1.2 m sucrose. After ultracentrifugation at 100,000 × *g*, 4°C for 2 h, the synaptosome fraction at the interface of 0.85/1.2 m sucrose was collected, diluted 6 times with 5 mm HEPES buffer, pH 7.4, and centrifuged at 80,000 × *g*, 4°C for 40 min. The pellet was resuspended with 200 μl 5 mm HEPES buffer, pH 7.4. Protein concentration was determined by Bradford (Bio-Rad) and adjusted to 0.75 μg/μl, then confirmed by running on a 10% stain-free SDS-PAGE system. All the buffer contains phosphatase inhibitor cocktails 2 and 3 (Sigma Millipore). The obtained synaptic membranes were subjected to trypsin digestion and iTRAQ reagent tagging.

##### Protein digestion and iTRAQ labeling

In three independent 8-plex iTRAQ experiments, we compared WT samples (*n* = 8) with *L7-PP2B* KO samples (*n* = 8) (two sets) and WT (*n* = 5) with α6-PP2B KO (*n* = 3, one set). The digestion and iTRAQ labeling of proteins in synaptic membrane fractions have been described previously (Li et al., 2007; Klychnikov et al., 2010). In short, for each sample, 75 μg of dried synaptic membranes was resuspended in 28 μl of 0.5 m triethylammonium bicarbonate buffer (Sigma Millipore), pH 8.5, containing 0.85% RapiGest (Waters). A 2 μl cleavage reagent (iTRAQ reagent kit, AB Sciex) was added and incubated at 55°C for 1 h, after which 1 μl of Cys blocking reagent (iTRAQ reagent kit, AB Sciex) was added and samples were vortexed for 10 min. Subsequently, 5 μg of trypsin (sequencing grade, Promega) was added and incubated 2 h at 55°C. The tryptic peptides were then tagged with iTRAQ reagents. After incubation for 3 h, the samples were pooled and acidified with 10% trifluoroacetic acid to pH 2.5-3. After 1 h, the sample was centrifuged, and the supernatant was dried in a SpeedVac. In each iTRAQ experiment, tissue from 4 WT and 4 KO were isolated (blinded for the experimenter) and tagged with 113 Da, 114 Da, 115 Da, 116 Da, 117 Da, 118 Da, 119 Da, and 121 Da reagents.

##### Two-dimensional liquid chromatography

The dried iTRAQ labeled sample was dissolved in 200 μl of loading buffer (20% ACN, 10 mm KH_2_PO4, pH 2.9), whereas 200 μl was injected into a strong cation exchange column (2.1 × 150 mm PolySΜLFOETHYL A column, PolyLC). Peptides were eluted with a linear gradient of 0-500 mm KCl in 20% ACN, 10 mm KH_2_PO_4_, pH 2.9, over 25 min at a flow rate of 200 μl/min. Fractions were collected at 1 min intervals and dried in a SpeedVac. SCX fractions were redissolved in 20 μl of 0.1% TFA, and fractionated by C18 nano-liquid chromatography (standard 2D-LC procedure).

##### MALDI-MS/MS

The sample was analyzed on an ABI 5800 proteomics analyzer (AB Sciex). Peptide collision-induced dissociation was performed at 1 kV; the collision gas was air. MS/MS spectra were each collected from 1500 laser shots. Peptides with the signal-to-noise ratio > 50 at the MS mode were selected for an MS/MS experiment; a maximum of 20 MS/MS was allowed per spot. The precursor mass window was 200 relatives to resolution (FWHM).

##### iTRAQ protein identification and quantitation

To annotate spectra, Mascot (Matrix Science) searches were performed against the SwissProt database (release November 2011) and the larger but more redundant NCBI database (release November 2011) using the GPS Explorer (AB Sciex version 3.6). MS/MS spectra were searched against mouse databases with trypsin specificity and fixed iTRAQ modifications at lysine residues and N-termini of the peptides. Mass tolerance was 150 ppm for precursor ions and 0.5 Da for fragment ions; one missed cleavage was allowed. The false discovery rate (percentage) for peptide identification was calculated using a randomized database. Protein redundancy in the result files was removed by clustering the precursor protein sequences at a threshold of 90% sequence similarity over 85% of the sequence length (Blastclust, version 20041205). Subsequently, all peptides were matched against the protein clusters; those that were matched to more than one protein cluster were not considered for protein identification and quantification, leaving only “unique” peptides in the analysis. Only proteins identified with at least two peptides with a CI ≥ 95% (AB Sciex, percentage) were considered identified; and of these proteins, only those with three or more quantifiable peptides in both iTRAQ experiments were included in subsequent quantitative analyses. Peak areas for each iTRAQ signature peak (m/z 113.1, 114.1, 115.1, 116.1, 117.1, 118.1, 119.1, 121.1) were obtained and corrected according to the manufacturers' instructions to account for the differences in the isotopic overlap. To compensate for the possible variations in the starting amounts of the samples, the individual peak areas of each iTRAQ signature peak were log2-transformed and normalized to the total peak area of the signature peak. Peptides with iTRAQ signature peaks of <2000 were not considered for quantification. Within each experiment, for each peptide, the peak area in each sample was mean-centered. Protein averages were calculated from these mean-centered peak areas of multiple peptides. Finally, the eight mutants and eight WT protein means of both experiments were used to calculate the average difference between WT and *L7-PP2B* KO mice. To assess whether this difference had occurred by chance because of the multiple testing problem or could be deemed significant, we calculated the permutation-derived false discovery rate (*q* value) using the Excel plug-in of the Significance Analysis of Microarrays program (Roxas and Li, 2008). The settings for the Significance Analysis of Microarrays program were as follows: two class unpaired, log_2_-scaled, *t* statistic, 1000 permutations, automatic estimation of s0 factor, and 10 *k-nearest* neighbors.

##### Phospho-proteomics protein extraction and digestion

Cerebellar P2 from 2 males and 1 female mouse were pooled to ensure enough yield of phosphopeptides after enrichment, using the same extraction buffer system as iTRAQ experiment. Protein concentrations were measured using the BCA assay (Thermo Fisher Scientific). Proteins were extracted by acetone precipitation at −20°C overnight. Samples were centrifuged at 8000 × *g* for 10 min at 4°C. The acetone was removed and the pellet allowed to dry. The protein pellet (∼4 mg protein) was dissolved in 1 ml 50 mm Tris/HCl, pH 8.2, 0.5% SDC; and proteins were digested with LysC (1:200 enzyme: protein ratio) for 4 h at 37°C. Next, trypsin was added (1:100 enzyme: protein ratio) and the digestion proceeded overnight at 30°C. Digests were acidified with 50 μl 10% formic acid (FA) and centrifuged at 8000 × *g* for 10 min at 4°C to remove the precipitated SDC. The supernatant was transferred to a new centrifuge tube. The digests were purified with C18 solid-phase extraction (Sep-Pak, Waters), lyophilized, and stored at −20°C.

##### Phosphopeptide enrichment

Phosphopeptide enrichment proceeded with some modifications to the method of Kettenbach et al. (2011) 4 mg lyophilized peptide digest was dissolved in 1 ml 50% ACN, 2 m. Lactic acid with 6 mg TiO_2_ beads (GL Sciences) and incubated on a rotator at room temperature for 2 h. Beads were washed twice with 2 m lactic acid/50% ACN and once with 4% FA in 50% ACN. Phosphopeptides were eluted twice with 150 μl of 50 mm K_2_HPO_4_, 1% pyrrolidine, acidified with 90 μl of 10% FA, and stored at −20°C. Tandem Mass Tagging labeling—Isobaric labeling of the enriched phosphopeptides was performed using the 10-plex tandem mass tag (TMT) reagents (Thermo Fisher Scientific) with some modifications to the method of Böhm et al. (2015). Phosphopeptides were loaded onto 20 mg C18 cartridges prepared in-house. The C18 cartridges were washed once with 1 ml 0.1% TFA and 2 times with 1 ml of 50 mm KH_2_PO_4_, pH 4.5. TMT reagents (0.8 mg) were dissolved in 10 μl of dry ACN and diluted with 200 μl 50 mm KH_2_PO_4_. This TMT solution was immediately loaded onto the column and labeling on column proceeded for 1 h at rrom temperature. Each of the 10 samples was labeled with a different TMT tag. After labeling, the column was washed twice with 1 ml 2% ACN/0.2% FA and the labeled peptides were eluted with 1 ml 50% ACN. TMT-labeled samples were pooled and lyophilized. High-pH and reversed phase HPLC—TMT labeled phosphopeptides were subjected to offline orthogonal high-pH and reverse phase fractionation. TMT labeled phosphopeptides were solubilized in 0.1% TFA and loaded onto a 20 mg PLRP-S cartridge made in-house. The cartridge was washed once with 1 ml 0.1% TFA and 3 times with 1 ml milliQ water. The peptides were eluted stepwise from the column with 0%, 5%, 10%, 15%, 25%, and 40% ACN/10 mm ammonium formate, pH 10. The 6 fractions were dried by vacuum centrifugation, and each fraction was reconstituted with 2% ACN/0.2% FA for nanoLC-MS/MS analysis.

##### Orbitrap lumos parameters

Mass spectra were acquired on an Orbitrap Lumos (Thermo Fisher Scientific) coupled to an EASY-nLC 1200 system (Thermo Fisher Scientific). Peptides were separated on an in-house packed 75 μm inner diameter column containing 50 cm Waters CSH130 resin (3.5 μm, 130 Å, Waters) with a gradient consisting of 2%-20% ACN, 0.1% FA over 150 min at 300 nl/min. The column was kept at 50°C in a NanoLC oven, MPI design (MS Wil). For all experiments, the instrument was operated in the data-dependent acquisition mode. MS1 spectra were collected at a resolution of 120,000 with an automated gain control (AGC) target of 2E5 and a maximum injection time of 50 ms. The most intense ions were selected for MS/MS, top speed method 3 s cycle time. Precursors were filtered according to charge state (2-7), and monoisotopic peak assignment. Previously interrogated precursors were dynamically excluded for 70 s. Peptide precursors were isolated with a quadrupole mass filter set to a width of 0.7 Th. When applying the MS3 method, ion trap MS2 spectra were collected at an AGC of 5E4, maximum injection time of 50 ms and CID collision energy of 35%. For Orbitrap MS3 spectra, the operation resolution was 60,000 with an AGC setting of 1E5 and a maximum injection time of 120 ms. The HCD collision energy was set to 65% to ensure maximal TMT reporter ion yield. Synchronous precursor selection was enabled at all times to include up to 10 MS2 fragment ions in the MS3 scan.

##### Data analyses of molecular work

Peak lists were automatically created from raw data files using the Proteome Discoverer 2.1 (Thermo Fisher Scientific) software. The Mascot search algorithm (version 2.2, MatrixScience) was used for searching spectra against the UniProt database (taxonomy: *Mus musculus*, version December 2016). The peptide tolerance was set to 10 ppm, and the fragment ion tolerance was set at 0.6 Da. A maximum number of 2 missed cleavages were allowed. TMT tags on peptide N-termini/lysine residues (229.162932 Da) and carbamidomethylation of cysteine residues (57.02,146 Da) were set as static modifications, while methionine oxidation (15.99 492 Da) and serine, threonine, and tyrosine phosphorylation (79.96 633 Da) were set as variable modifications. The target FDR for both PSMs and peptides was set at 0.01. Only peptides marked “high confidence” were taken into account for further analysis. Proteins were marked with “high confidence” when they fulfilled the requirement for an FDR = 0.01. The co-isolation threshold was set at 75% and the minimum signal-to-noise ratio at 10. For TMT quantification, a 0.01 Th window centered on the theoretical m/z value of each reporter ion was queried for the nearest signal intensity. Reporter ion intensities were adjusted to correct for the isotopic impurities of the different TMT reagents.

##### Electron microscopy

To assess the potential influence of a lack of PP2B on the postsynaptic density at the PF inputs to PCs beyond the age of 5 months (Schonewille et al., 2010), we investigated these synapses of lobules 3 and 9 in 6- to 8-month-old *L7-PP2B KO* mice and WT littermates at the EM level (for details on EM procedures, see De Zeeuw et al., 1998). In short, the mice were deeply anesthetized with Nembutal and perfused transcardially with 0.9% saline in 0.1 m cacodylate buffer at pH 7.4, followed by 5% glutaraldehyde in the same buffer. The cerebellum was removed, kept in fixative for 2 h, and cut transversely with a Vibratome into 70 µm sections. The sections were osmicated with 1.5% osmium tetroxide in 0.1 m PB, pH 7.3, during 40 min at 45°C, rinsed in distilled water (4 times), block-stained in 2% aqueous uranyl acetate for 30 min at room temperature, dehydrated in dimethoxypropane, and embedded in araldite. Areas of lobules 3 and 9 were selected in semithin sections, and ultrathin sections were cut from the selected tissue blocks. The grids with the sections were counterstained with uranyl acetate and lead citrate, and examined in a Talos 120 (Thermo Fisher Scientific) EM operating at 80 kV. In the double-blind analyses, we first identified the PF to PC synapses according to the criteria described by Palay and Chan-Palay (1974), which included size of the presynaptic and postsynaptic structure, as well as density and shape of the vesicles. We subsequently measured the thickness and length of the postsynaptic density (done with Fiji) and calculated the relative thickness (i.e., thickness divided by length) and area (i.e., thickness times length). Statistical analysis was done as described previously (De Zeeuw et al., 1998).

##### Cell cultures

PCs were isolated from E17-E19 mice embryos following a method previously described (Tabata et al., 2000) with slight modifications. Briefly, the cerebella were dissected in ice-cold HBSS supplemented with 20 µg/ml gentamicin (both from Invitrogen), then incubated with 10 U/ml papain (Sigma Millipore) and 2.5 U/ml DNase I (Roche Diagnostic) and 4 mm MgCl_2_ (Sigma Millipore) at 33°C for 20 min. The cerebella were titrated in HBSS with 2.5 U/ml DNase I and 4 mm MgCl_2_ and were filtered with 200 µm Nylon mesh (Millipore). After washing twice in HBSS, the cells were plated on precleaned, poly-ornithine (500 µg/ml, Sigma Millipore) coated 1.5H glass-bottomed slide (Ibidi) at the density of 1.2 × 10^6^ cells/cm^2^. For tracking experiments, the cells were transfected before plating with L7-mCherry or -GFP (a gift from J Hammer III) (see Wagner et al., 2011) using Nucleofector 4D (Lonza Walkersville) according to manufacturer's protocol. The culture medium contained PNBM neural basal medium (Lonza Walkersville), GS21 neural supplement (1:50, Globalstem), 5 µg/ml gentamicin, and 2 mm Glutamax (Invitrogen); half-volume of the medium was changed once a week and the day before the experiment.

##### Electrophysiology

Acute sagittal slices (250 μm thick) were prepared from the cerebellar vermis in ice-cold slicing medium (in mm): 240 sucrose, 2.5 KCl, 1.25 Na_2_HPO_4_, 2 MgSO_4_, 1 CaCl_2_, 26 NaHCO_3_, and 10 D-glucose, bubbled with 95% O_2_ and 5% CO_2_. Slices were incubated in ACSF containing (in mm): 124 NaCl, 2.5 KCl, 1.25 Na_2_HPO_4_, 2 MgSO_4_, 2 CaCl_2_, 26 NaHCO_3_, and 10 D-glucose, bubbled with 95% O_2_ and 5% CO_2_ at 34.0°C for 30 min, and kept at room temperature before use. PCs were recorded using intracellular solution contains the following (in mm): 120 K-gluconate, 9 KCl, 10 KOH, 3.48 MgCl_2_, 4 NaCl, 10 HEPES, 4 Na_2_ATP, 0.4 Na_3_GTP, and 17.5 sucrose, pH 7.25 and Osm 295. The sEPSCs were analyzed using Mini Analysis version 6.0.3.

##### dSTORM

The cerebellar culture was fixed with 4% PFA/4% sucrose in PBS on DIV 21 at room temperature for 10 min, washed 3 times with PBS containing 10 mm Tris-PBS, pH 7.4. Slides were blocked in 10% horse serum with 0.1% Triton X-100 (Sigma Millipore) for 1 h at room temperature (for surface GluR2 staining, Triton X-100 was excluded), then incubated with primary antibodies at 4°C overnight. After washing with DPBS for 3 × 5 min, the secondary antibodies (6.7 μg/ml, Thermo Fisher Scientific or The Jackson Laboratory) were added and incubated for 1 h at room temperature. Then slides were washed with DPBS for 3 × 5 min, postfixed with 4% PFA at room temperature for 10 min, washed 3 times with Tris-PBS, and stored at 4°C with 1:15K diluted 0.1 µm TetraSpeck Microspheres (Thermo Fisher Scientific). Imaging buffer contained 10% glucose (w/v), 0.56 mg/ml glucose oxidase, 34 μg/ml catalase, and 25 mm MEA in 50 mm Tris HCl, pH 8.0, 10 mm NaCl, and imaging was done with the use of a Carl Zeiss Elyra microscope, using ZEN software for the analysis. Alexa647 for dSTORM samples was used within 2 weeks. During imaging, no free-floating fluorophore was observed. The analysis was done with custom-written scripts in Fiji and SMoLR (R). Briefly, the spine heads (i.e., the ROI) were picked objectively with Fiji, the imported localizations files were analyzed blindly in R, and clusters were extracted from the localization data using DBSCAN (Ester et al., 1996). After removal of background, the sizes of nanoclusters were plotted in R using ggplot2 (Wickham, 2009). The size of the clusters was calculated as the SD along major axis.

##### Live cell imaging

The single-molecule tracking experiments were done on DIV 21-23 with a Carl Zeiss Elyra PS1 microscope, integrated with an LSM 780 confocal microscope. We used a 100×-1.49oilα Plan Apochromat DIC objective, and the imaging solution contained the following (in mm): 135 NaCl, 3 KCl, 2 CaCl_2_, 2 MgCl_2_, 20 sucrose, and 10 HEPES, pH 7.25, at 37°C. The targeted PCs were identified by the expression of L7-mCherry and their morphology, and a confocal *z* stack was obtained for later reference. The imaging started immediately after adding the primary antibody, which was labeled with Alexa-488 at a concentration of 1:1500. The images were obtained with an EMCCD iXon DU897 (Andor) at 30 Hz for 5000 frames with the use of HiLo imaging. For analysis, the appropriate confocal image was used to align with the time-lapse images, and the tracking within the ROI was done in Fiji using the SOS plugin (Yao et al., 2017). Tracks shorter than 10 frames were excluded from analysis. Mean square displacements (MSD) were estimated for the individual tracks, and apparent diffusion constants were subsequently estimated by fitting a linear curve to the mean square displacement as a function of time.

## Results

### Selective inhibition of phosphatase activity of PP2B only partially impairs cerebellar learning

*L7-PP2B KO* mice, in which PP2B is specifically knocked out from PCs, have been shown to suffer from impaired cerebellar learning, such as adaptation of the VOR (Schonewille et al., 2010). We evaluated to what extent these deficits can be explained by loss of only the phosphatase activity of PP2B during learning. To this end, WT mice (*n* = 8) were systemically injected with the selective PP2B inhibitor, FK506 (Butcher et al., 1997; Pardo et al., 2006) for 5 consecutive days during which their eye movements were measured. Pharmacological inhibition of PP2B by FK506 might in principle be expected to induce a comparable blockage of the enzymatic activity of PP2B as a genetic deletion of PP2B, since both result in a similar hyperphosphorylation of Ser778 in Dynamin1 (Clayton et al., 2009; Cottrell et al., 2013). Levels of eye movement performance and learning were assessed, and compared with those in *L7-PP2B KO* mice (*n* = 7) and WT controls (*n* = 7) that received injections with the vehicle (10% DMSO, 10% ethanol in 0.9% saline). The baseline motor performance, as measured by visually guided vestibular-ocular reflex (VVOR) was similar across the three groups (Fig. 1*A*). We subsequently probed the level of cerebellar learning in these mice with a visuo-vestibular gain-decrease training paradigm (Schonewille et al., 2010). Whereas the groups injected with FK506 and vehicle-only learned equally well in this gain-decrease paradigm (*p* > 0.9 without Bonferroni correction; prelearning vs postlearning), *L7-PP2B KO* mice were unable to learn the same paradigm (*p* = 0.0003 with Bonferroni correction; unless stated otherwise, all significant differences described below were subjected to the same correction for multiple comparisons) (Fig. 1*B*). Subsequent VOR phase-reversal training over 5 consecutive days caused a prominent reversal of eye movement direction in the animals injected with vehicle-only and some learning in the animals injected with FK506 but failed to induce phase-reversal in the *L7-PP2B KO* mice (*p* < 0.0001) (Fig. 1*C*). Thus, although chronic pharmacological inhibition of the enzymatic function of PP2B resulted in a deficit in phase-reversal learning, the level of learning was significantly better than that in *L7-PP2B KO* mice (*p* < 0.0001) (Fig. 1*C*). It should be noted that *L7-PP2B KO* mice injected with FK506 (*n* = 3; tested for control) did not show any significant learning, just like the *L7-PP2B KO* mice injected with vehicle only. Given the difference in behavioral learning between the animals injected with FK506 and the *L7-PP2B KO* mice, we set out to investigate to what extent PP2B may also exert functions other than regulating protein dephosphorylation.

10.1523/JNEUROSCI.1741-20.2021.f1-1 Figure 1-1 Antibody list.xlsx: Antibody information used in this study Download Figure 1-1, XLSX file.

### Ablation of PP2B reduces PSD protein and receptor levels in PCs

**Figure 2.**
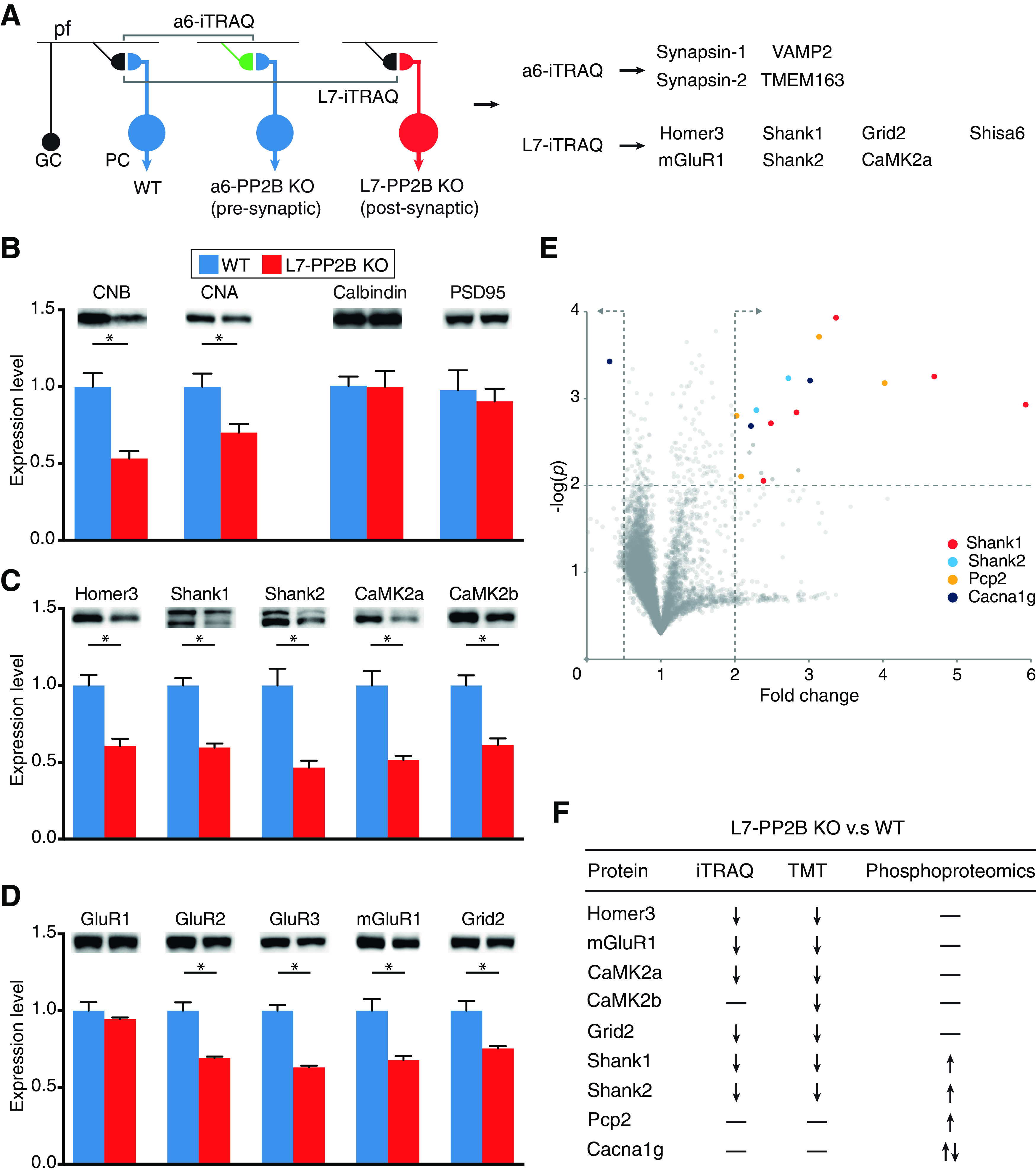
Specific presynaptic and postsynaptic KO of PP2B at PF-PC synapses resulted in downregulation of presynaptic and postsynaptic proteins, respectively. ***A***, Scheme of cerebellar cortex circuity and iTRAQ experimental setup (left) and list of significantly downregulated proteins in α6-PP2B (presynaptic KO) and L7-PP2B (postsynaptic KO) synaptosomes identified by 8-plex iTRAQ (*n* = 4:4 mice per run, repeated twice) (right). The presynaptic axon terminals of WT and PP2B KO GCs, which are making synaptic contact with the spine heads of PCs, are represented in black and green, respectively. WT PCs and PP2B KO PC (both cell bodies and spine heads) are represented in blue and red, respectively. Arrows leaving the PCs indicate that they form the sole output of the cerebellar cortex, with their axons traversing toward the cerebellar nuclei. ***B***, Immunoblots of *L7-PP2B KO* and WT littermates synaptosomes (*n* = 6:6 mice, 10-12 PND). Whereas CNB and CNA confirmed successful KO of PP2B (*p < 0.05), calbindin and PSD95 signals suggest that the number of PCs and synapses are not affected by knocking out PP2B, respectively (*p* = 0.9009, *p* = 0.2413, unpaired parametric *t* test). ***C***, Immunoblots showing that multiple PSD proteins are downregulated in *L7-PP2B KO*. ***D***, Immunoblots confirming that several glutamate receptor subunits are significantly downregulated in *L7-PP2B KO*. Error bars indicate ± SEM (*p < 0.05). ***E***, Phospho-proteomic volcano plot reveals changed detection of protein/phosphor sites in L7-PP2B mice (P2, 10-plex TMT, *n* = 5 × 3 mice:5 × 3 mice, 10-12 PND). The *x* axis and *y* axis indicate fold change and log(*p* value), respectively. Area of upper quadrant on the right shows proteins with a phosphorylation fold change > 2 (unpaired *t* test, *p* < 0.01) following comparison of WT and L7-PP2B mice P2 fractions. For example, only 1 site of Cacna1g is less phosphorylated in L7-PP2B mice, whereas others are more phosphorylated. Moreover, a number of other sites of other proteins, such as Shank1, Shank2, and pcp2, are only more phosphorylated. ***F***, Summary table of changes in protein levels as observed following iTRAQ (8-plex), TMT (10-plex) for total proteins, and for phospho sites determined by phospho-proteomics.

To clarify the effects of genetic deletion of PP2B at the level of protein levels in the PF to PC synapse (De Zeeuw and ten Brinke, 2015), we used a quantitative iTRAQ proteomic analysis (see Materials and Methods) of the molecular layer of either *L7-PP2B KO* or *a6-PP2B KO* mice, in which PP2B was selectively ablated from PCs (Schonewille et al., 2010) or GCs (Galliano et al., 2013), respectively. Given that GC axons and PC spines are the presynaptic and postsynaptic components of the PF to PC synapses, both of which might be subject to PP2B regulation (Schonewille et al., 2010; Silverman-Gavrila et al., 2013), this approach enabled the comparison of protein composition in which PP2B was selectively deleted from the presynaptic or postsynaptic compartment of the PF-PC synapse (Fig. 2*A*). Quantitative iTRAQ analysis of the synaptic membrane fraction of the L7-PP2B mice revealed a statistically lower level of seven proteins, including five PSD-enriched proteins (Homer3, Shank1, Shank2, αCaMKII, and Shisa6) and two postsynaptic receptors (mGluR1, Grid2) (Fig. 2*A*; Extended Data Fig. 2-1). Because of the stringent criteria applied to the iTRAQ analysis (see Materials and Methods), these results are likely to represent a fraction of the overall alterations in the synapse. The changes in protein levels of most of these proteins were confirmed by immunoblotting of the L7-PP2B versus WT PF-PC synaptic membrane fraction in three independent experiments (Fig. 2*B-D*). The finding that the changes in the scaffold proteins Shank1, Shank2, and Homer3 align closely with those of CNA and CNB agrees well with the fact that the PF to PC synapse is the most numerous in the cerebellar cortex (Galliano et al., 2013) and raises the possibility that these proteins interact with each other (see also below). In addition, lower levels of βCaMKII, GluR2, and GluR3 were found in the L7-PP2B mice, presumably reflecting the relatively high level of sensitivity of immunoblotting. Interestingly, none of these proteins was significantly downregulated in the α6-PP2B KO mice (Extended Data Fig. 2-2). Instead, the iTRAQ analysis of the α6-PP2B KO mice showed statistically altered expression of other presynaptic proteins, such as Synapsin1/2, VAMP2, and TMEM163 (Fig. 2*A*), thereby highlighting the specificity of the postsynaptic impact of the PP2B deletion in the L7-PP2B mice.

10.1523/JNEUROSCI.1741-20.2021.f2-1 Figure 2-1 L7-PP2B iTRAQ.xls: iTRAQ results comparing *L7-PP2B KO*:WT Download Figure 2-1, XLS file.

10.1523/JNEUROSCI.1741-20.2021.f2-2 Figure 2-2 α6-PP2B iTRAQ.xls: iTRAQ results comparing α6-PP2B KO:WT Download Figure 2-2, XLS file.

**Figure 3.**
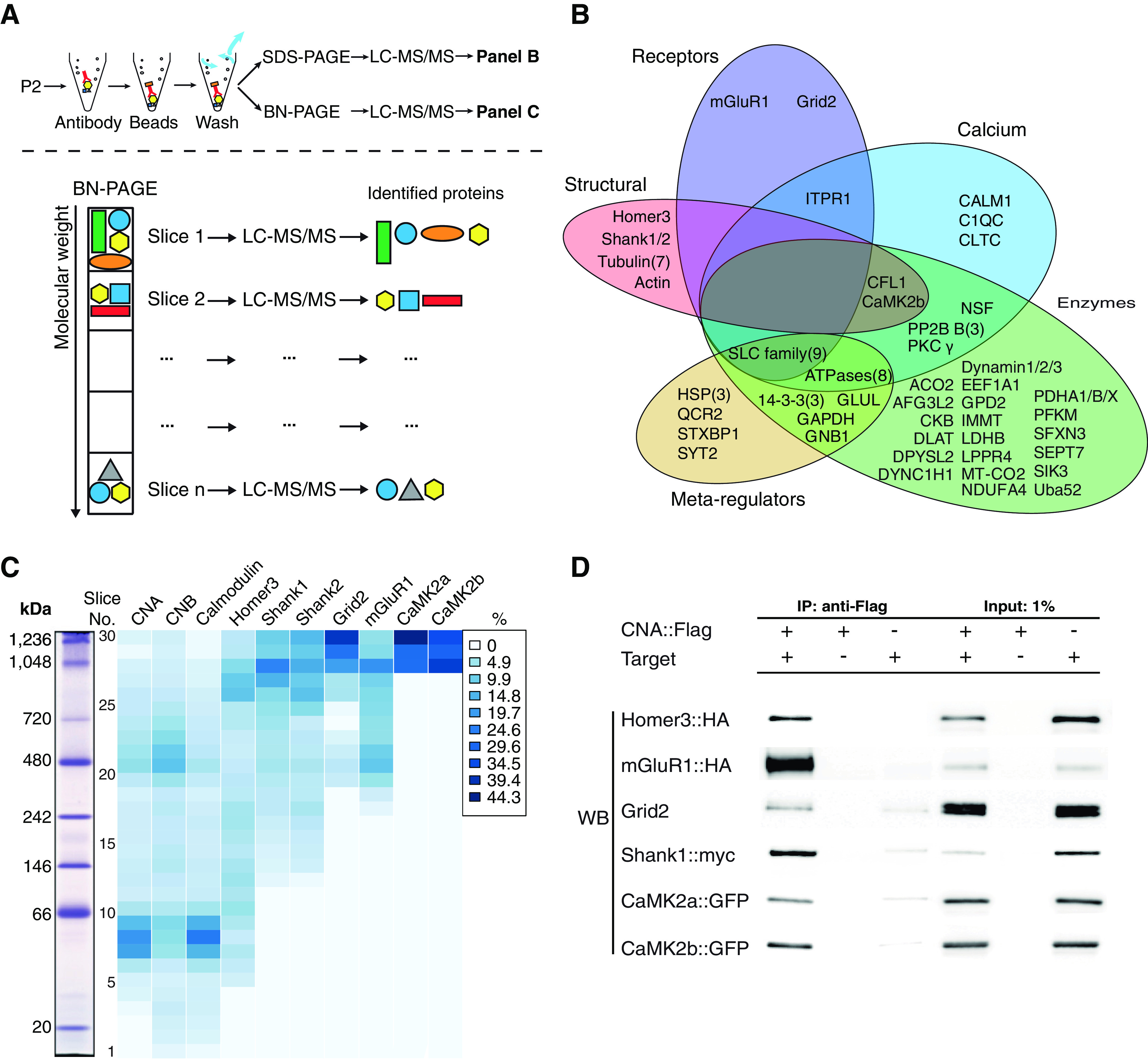
PP2B stably and directly interacts with multiple PSD proteins. ***A***, Illustration of IP-MS/MS workflow. Top, IP-MS/MS for cerebellar PP2B in WT mice P2 fractions. Bottom, Scheme of IP-BN (blue native)-PAGE-MS/MS. ***B***, IP-MS/MS result. Proteins identified as PP2B interactors. These proteins were identified in IP with 2 PP2B antibodies, and at least twice for each antibody (see Materials and Methods; Extended Data Figure 3-1). Proteins were manually grouped by their known overall functions. The number in the brackets indicates the number of proteins belonging to that group. Only one protein (C1QC) from the IP-MS/MS result was not included in the graph. ***C***, The result of IP/BN-PAGE/MS from WT cerebellar P2 fractions. Detection of different proteins is presented in columns, fractions analyzed by MS are presented in rows, and colors are coded with normalized iBAQ value (in percentage of total). CNA, CNB, and calmodulin are well colocalized. The interactors of CNA, including CaMK2, Homer3, Shank1, Shank2, Grid2, and mGluR1, are enriched in the high-molecular weight fractions; proteins may form multiple complexes. ***D***, HEK293 cell co-IP experiments confirming direct interactions with the candidate proteins from cerebellum IP, Homer3, mGluR1, Shank1, and CaMK2b, as illustrated by the enrichment of the first column compared with the second and third column. This figure is constructed by horizontally combining Western blots from different individual target proteins.

10.1523/JNEUROSCI.1741-20.2021.f2-3 Figure 2-3 phosphor-proteomics and TMT.xlsx: phosphor-proteomics and TMT results comparing *L7-PP2B KO*:WT Download Figure 2-3, XLSX file.

These results raise the question to what extent the observed lower protein levels in the *L7-PP2B KO* mice can be explained by an altered level of phosphorylation. We therefore compared the level of synaptic phosphopeptides in a synaptic membrane fraction between the *L7-PP2B KO* and their WT littermates (see Materials and Methods). Using TMT, another isotope labeling-based quantitative proteomic analysis method, next to the phospho-proteomics enables us to be clear about the changes of a post-translational added/removed phosphate group that without TMT detection could have resulted from having more/less protein. The TMT results mostly confirmed our iTRAQ findings (Fig. 2*F*). A total of 37,752 phosphopeptides were obtained and quantified; these phosphopeptides were part of a total of 1881 proteins (Extended Data Fig. 2-3). We found significantly (*p* < 0.01, FDR 0.01) elevated phosphorylation levels for several phosphorylation sites of Shank1, Shank2, pcp2, and Cacna1g (Fig. 2*E*; Extended Data Fig. 2-3). The phosphorylation states of most of the proteins that showed reduced levels in the *L7-PP2B KO* (e.g., Homer3, mGluR1, αCaMKII, and Grid2) were not significantly increased (Fig. 2*E*, top right corner; Extended Data Fig. 2-3). Our data suggest that deleting PP2B may regulate the level of various postsynaptic proteins without necessarily affecting their net-phosphorylation level.

### PP2B directly interacts with multiple PSD proteins

To explore how PP2B deletion could affect the synaptic protein levels at the PF-PC synapse, we used immunoprecipitation of PP2B to capture PP2B interactors from the P2+microsome fraction of cerebella. We subsequently determined their identity using high-resolution liquid chromatography/mass spectrometry (LC-MS/MS) (Fig. 3*A*, top). Of all proteins detected in this immunoprecipitation, 78 were considered stable PP2B interactors (see Materials and Methods). We then performed an Ingenuity Pathway Analysis, which divided these proteins into five partially overlapping groups of molecular function (Fig. 3*B*; Extended Fig. 3-1). These included receptors (Group 1: e.g., mGluR1 and Grid2), calcium-regulated proteins (Group 2: e.g., calmodulin, βCaMKII, and calcium-dependent ATPases), enzymes (Group 3: e.g., Dynamin, PLPPR4, and DLAT), meta-regulators (Group 4: e.g., HSP3, SYT, and 14-3-3(3)), and structural proteins (Group 5: e.g., Homer3, Shank1/2, and Actin). These results indicate that PP2B can potentially interact with proteins belonging to different functional groups in PF-PC synapses.

10.1523/JNEUROSCI.1741-20.2021.f3-1 Figure 3-1 L7-PP2B IP.xlsx: IP-MS result with PP2B antibodies from WT Download Figure 3-1, XLSX file.

10.1523/JNEUROSCI.1741-20.2021.f3-2 Figure 3-2 BN-PAGE.xlsx: IP-BN-PAGE-MS result with PP2B antibody from WT Download Figure 3-2, XLSX file.

To investigate the composition of PP2B-containing protein complexes in their native binding states in PF-PC synapses, we next performed blue-native PAGE(BN)-LC-MS/MS analysis on the PP2B immunoprecipitated samples. PP2B containing complexes were separated by mass on a blue-native gel and subsequently divided into 30 slices. Each slice was processed and analyzed for protein content by mass spectrometry separately (Fig. 3*A*, bottom; Extended Data Fig. 3-2). The PP2B subunits, CNA and CNB, were present in the BN gel throughout all molecular weights, demonstrating the mass diversity, and thus molecular diversity of PP2B-containing complexes (Fig. 3*C*). Two prominent peaks were observed in the CNA and CNB distribution; these peaks emerged at ∼60 and ∼480 kDa, respectively. These peaks likely reflect PP2B itself and the PP2B-interacting protein complexes, respectively. Interestingly, whereas calmodulin tightly migrated with the lower molecular weight form of PP2B complexes (∼60 kDa), mGluR1 migrated more closely with the higher molecular weight complex (∼480 kDa). Homer3, Shank1, Shank2, Grid2, αCaMKII, and βCaMKII were all detected predominantly in protein complexes appearing at higher molecular weights, ranging from ∼700 to ∼1250 kDa, indicating multiprotein assemblies. To further assess whether PP2B can directly interact with each of these interacting proteins, we coexpressed PP2B with 6 candidate proteins in HEK293 cells and tested their binding using IP. Because native PP2B is also present in HEK293 cells, exogenous CNA was labeled with a flag tag. Except for Shank2, all tested PSD proteins were confirmed to bind to the CNA subunit of PP2B (Fig. 3*D*). These data demonstrate that PSDs of PF-PC synapses contain three major PP2B-containing complexes, one with a low molecular weight consisting of PP2B and calmodulin, one with an intermediate weight interacting with mGluR1, and several likely minor multiprotein PP2B complexes with high molecular weights.

### PSDs of *L7-PP2B KO* PF-PC synapses show reduced relative thickness

**Figure 4.**
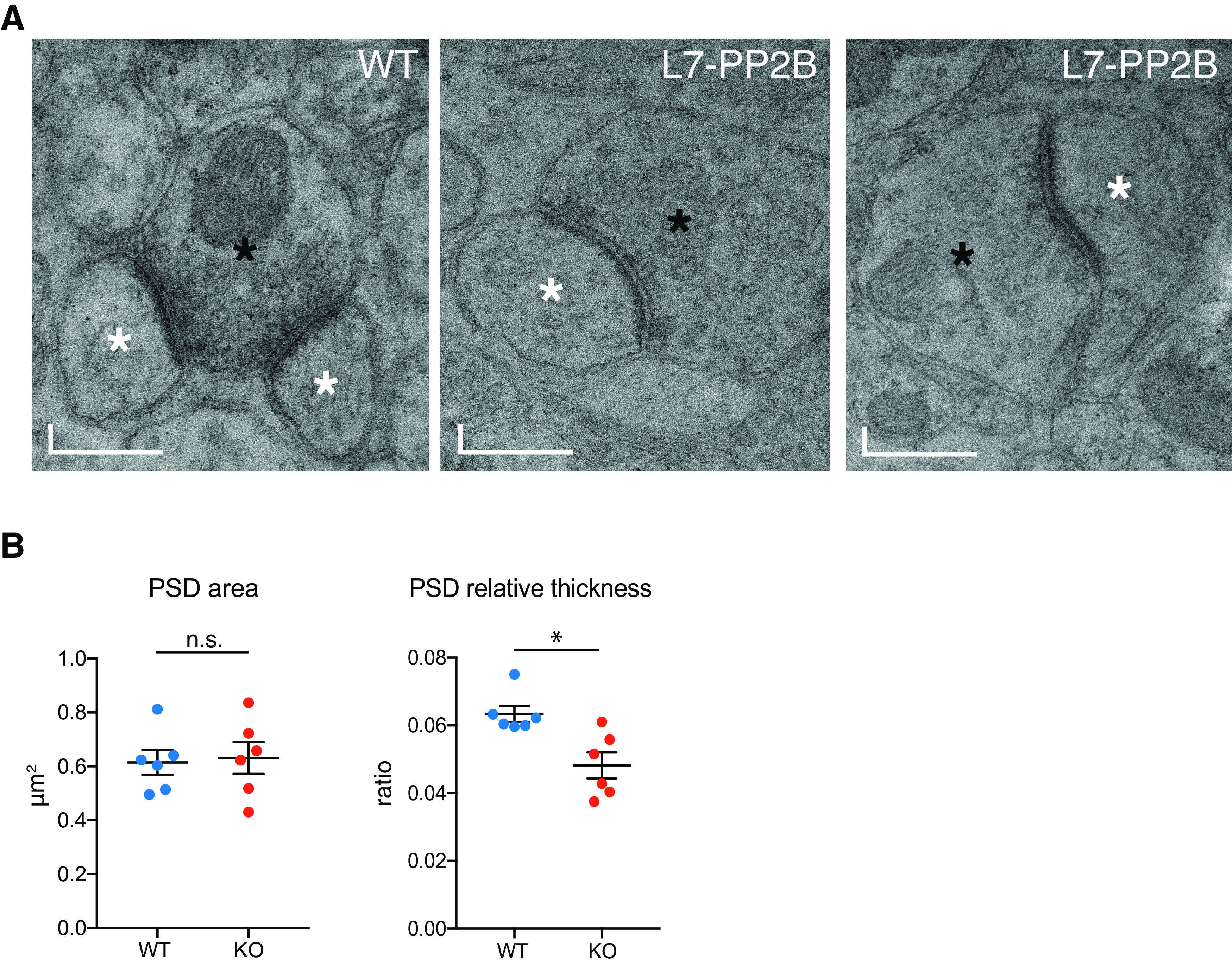
Ultrastructure of PF-PC synapses. ***A***, Electron micrograph of a PF terminal contacting two PC spines in WT (left) or a single spine in *L7-PP2B KO* mice (middle, right). Black and white asterisks indicate PF terminals and PC spines, respectively. Scale bars: left, 0.3 µm; middle, 0.4 µm; right, 0.4. ***B***, Quantification of average area and relative thickness (ratio of thickness per length) of PSDs for WT and L7-PP2B mice (25 synapses per lobule per mouse). *p* = 0.699 and *p* = 0.015 for area and relative thickness, respectively (Mann–Whitney test). Error bars indicate ± SEM, *p < 0.05.

Since PP2B interacts with several important PSD proteins (Figs. 2, 3), we investigated to what extent the ultrastructure of PF-PC synapses is affected by knocking out PP2B. We therefore measured the parameters of PSDs of PF-PC synapses in tissue blocks of lobules 3 and 9 from 6- to 8-month-old *L7-PP2B KO* mice (*n* = 6) and WT controls (*n* = 6). The area of the PSDs in the *L7-PP2B KO* mice, as measured in 2D sections, was not significantly different from that in WT littermates (*p* = 0.485; Mann–Whitney test; Fig. 4*A*). However, the relative thickness, measured by the ratio of PSD thickness to PSD length, was significantly lower in the *L7-PP2B KO* mice (*p* = 0.015; Mann–Whitney test; Fig. 4*B*). These results suggest that, whereas the total area of postsynaptic structure is maintained, the shape of the PSD is relatively thin across the length of the synaptic membrane in the absence of PP2B.

### PP2B differentially affects distribution of its PSD interactor proteins

**Figure 5.**
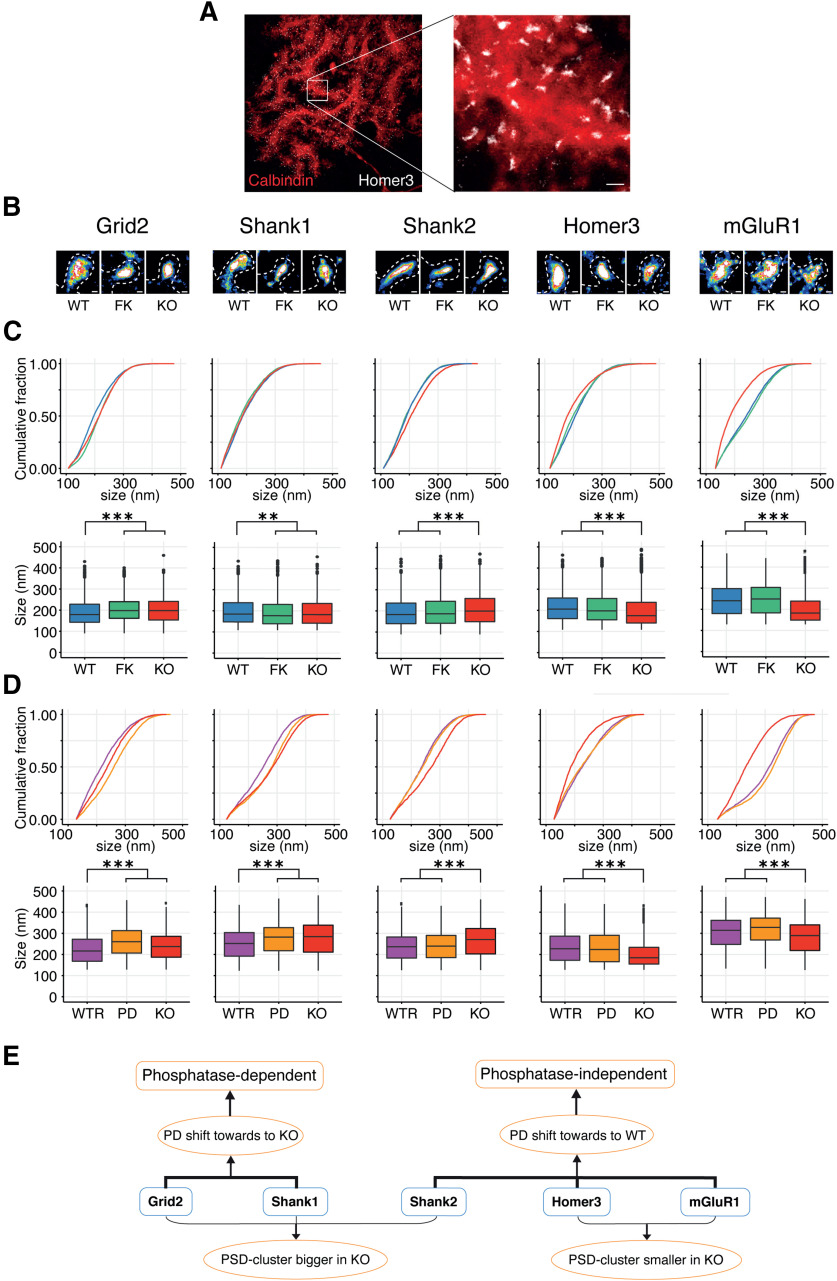
Cluster size of PP2B-PSD interactors as revealed with dSTORM. ***A***, Example of dSTORM aligned with confocal image of a cultured PC, illustrated with Homer3 dSTORM and calbindin staining. Scale bar: right, 400 nm. ***B***, Example of dSTORM images of cultured PC spines immunostained with PP2B PSD interactors. Scale bars, 100 nm. ***C***, Top and bottom, Cumulative and box plots, respectively. In the box plots, we compared WT (blue), WT with pharmacologically long-term blockage of phosphatase activity of PP2B by FK506 (FK, green), and KO (red). **p < 0.01; ***p < 0.001; Dunn test with Bonferroni correction. ***D***, Same as in ***C***, but for comparisons of KO infected with AAV encoding WT-PP2B (WTR; see Fig. 6*A*), KO infected with AAV encoding enzyme-dead PP2B (PD; see Fig. 6*A*), and KO infected with empty AAV (KO). ***E***, A model summarized from the results above. The cluster sizes of Homer3, mGluR1 were smaller in the PP2B KO, whereas Shank2, Shank1, and Grid2 showed bigger cluster sizes in PP2B KO. Moreover, clusters of Homer3, mGluR1, and Shank2 were rescued to WT level following expression of enzyme-dead PP2B in KO cells, indicating their regulation by PP2B via a phosphatase-independent manner. Instead, the cluster size of Shank1 and Grid2 did depend on PP2B phosphatase activity, as the enzyme-dead expression led to the same level as in *L7-PP2B KO* mice.

To determine to what extent PP2B has a role in controlling the distribution of its PSD interactors at the nanometer level, we investigated the densities of the five PSD proteins that interacted most prominently with PP2B (i.e., Shank1, Shank2, Grid2, Homer3, and mGluR1). Using super resolution direct stochastic optical reconstruction microscopy (dSTORM) (Huang et al., 2010), individual spines were extracted and a localization profile of the largest nanoclusters within that spine was analyzed (Fig. 5*A*). The Homer3 and mGluR1 nanoclusters in PCs derived from *L7-PP2B KO* mice were smaller than those from WTs, whereas the nanocluster sizes of Shank1, Shank2, and Grid2 were increased in the PP2B KO PCs (Fig. 5*B*,*C*). These results imply that the localization of Shank1, Shank2, and Grid2 proteins is more spread out in the absence of PP2B. We next asked to what extent these alterations were induced by a lack of phosphatase activity. Cultured WT PCs were treated with FK506 (2 μm) for 18 d (renewed every 3 d) to inhibit the phosphatase activity of PP2B. This inhibition shifted the cluster sizes of Grid2 and Shank1 obtained in WT PCs toward those in *L7-PP2B KO* PCs, whereas the cluster sizes of Shank2, Homer3, and mGluR1 remained at WT level. These data indicate that the synaptic localization of Shank2, Homer3, and mGluR1 does not depend on the phosphatase activity of PP2B (Fig. 5*B*,*C*).

To confirm that the nonenzymatic role of PP2B contributes to changing the densities of some of the proteins within the PSD, we investigated the effects of reexpressing enzyme-dead PP2B (referred to as PD) (Oliveria et al., 2012) in *L7-PP2B KO* PCs. Reexpressing PD PP2B in these KO cells restored the protein densities of only Shank2, Homer3, and mGluR1 to WT levels, whereas reexpressing native PP2B, which exerts both the nonenzymatic and enzymatic function, reinstated the size of the densities of all five synaptic proteins (Fig. 5*D*). These data highlight the differential impact of PP2B on the distribution of PSD proteins and indicate that the synaptic expression of Shank2, Homer3, and mGluR1 may be regulated by a PP2B-related mechanism that does not require its activity as a phosphatase (Fig. 5*E*).

### AMPAR mobility depends on PP2B phosphatase activity

**Figure 6.**
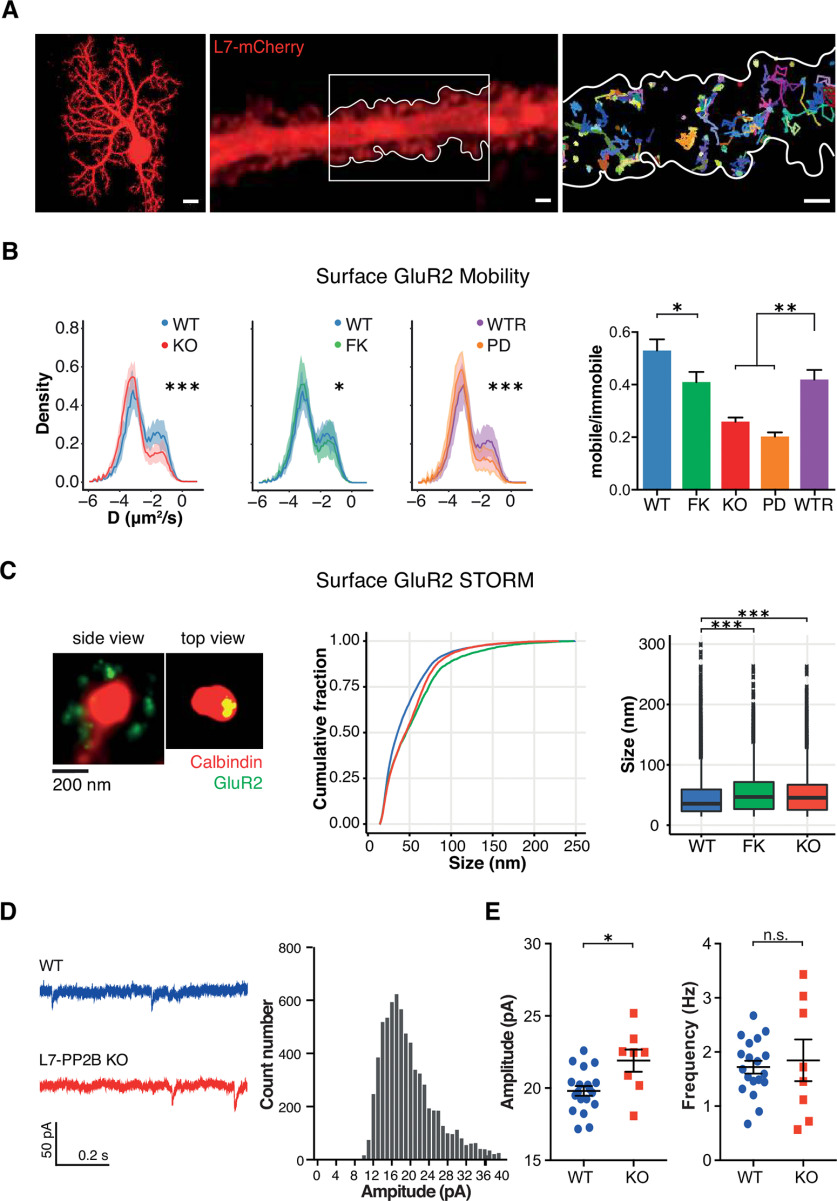
Single-molecule tracking of GluR2 in PC. ***A***, Left, Example of a cultured PC (DIV 21). Scale bar, 10 μm. Middle, Example of a piece of PC dendrite visualized by transfection of L7-mCherry. Scale bar, 1 μm. Right, Example of surface GluR2 tracks from the inset in the middle panel. Scale bar, 1 μm. ***B***, The surface GluR2 tracking results under different conditions, which are summarized in the histogram on the right (color coding at *x* axis applies for all panels). WT PCs show a larger percentage of mobile GluR2 than the FK and PP2B KO cells. Shades and error bars indicate SEM. Whereas the expression of WT PP2B in KO cells rescued the mobility (WTR, purple), the expression of enzyme-dead PP2B in KO cells (PD, orange) led to the same level of mobility as PP2B KOs (one-way ANOVA with Tukey's multiple comparisons, WT vs FK *p* = 0.0218; WT vs KO *p* < 0.0001; WT vs WTR *p* = 0.1232; WTR vs KO *p* = 0.0008; WTR vs PD *p* < 0.0001). ***C***, The surface GluR2 STORM in WT, FK, and KO cultured PCs. Left, Two examples of surface GluR2 dSTORM on a PC spine: one from the side and one from top. Middle, right, Cumulative and box plots, respectively. Dunn test with Bonferroni correction: WT versus FK *p* < 0.0001, WT versus KO *p* < 0.0001. ***D***, ***E***, mEPSCs recorded from acute slices of WT and *L7-PP2B KO* mice. ***D***, Left, Traces represent example traces of mEPSC recordings. Right, Histogram represents the mEPSC amplitude from all recordings. Bin size = 1 pA. ***E***, The amplitude of the mEPSCs is increased in the FK-treated PCs as well as the *L7-PP2B KO* PCs (Mann–Whitney test, *p* = 0.019, *n* = 19: 8 cells, WT:KO, from 3 pairs of mice), whereas their frequencies appear unaffected (Mann–Whitney test, *p* = 0.979). *p < 0.05; **p < 0.01; ***p < 0.001.

Given that the function of synapses is affected by the lateral mobility of their glutamate receptors (Heine et al., 2008; Penn et al., 2017), we examined to what extent the mobility of GluR2 was affected by pharmacological inhibition or genetic ablation of PP2B in cultured PCs. We performed single-molecule tracking for the ionotropic glutamatergic receptor subunit, GluR2 (i.e., the main receptor subunit present in PCs) (Tu et al., 1998; Chung et al., 2003; Gutierrez-Castellanos et al., 2017) (Fig. 6*A*). The overall mobility of this glutamate receptor subunit was similar to the ones observed in cultured hippocampal neurons (Constals et al., 2015). Inhibition of PP2B phosphatase activity in WT PCs with FK506 significantly reduced the receptor mobility of GluR2 (*p* = 0.02) (Fig. 6*B*). For control, we tested whether reexpressing PP2B in the *L7-PP2B KO* PCs could rescue the deficits in receptor mobility. While reexpressing WT (native), PP2B successfully rescued the mobility deficit of the AMPA receptors, it was not rescued by reintroducing the phospho-dead PP2B variant (PD, Fig. 6*B*). These results suggest that control of the lateral mobility of glutamate receptors depends on the phosphatase activity of PP2B.

Yet, the function of synapses is affected not only by the lateral mobility of glutamatergic receptors, but also by their total surface expression (Penn et al., 2017). Since the surface expression of AMPARs is regulated by their phosphorylation state and related to the level of LTD and LTP induction (Schonewille et al., 2010; Diering and Huganir, 2018), and since we found that a lack of PP2B phosphatase activity reduces the synaptically expressed GluR2 subunits (Fig. 2*D*), we set out to investigate the impact of PP2B on the distribution of surface GluR2 in PCs. When we inhibited PP2B phosphatase activity in WT PCs with FK506, we observed a consistent increase in the size of surface GluR2 clusters similar to that in PCs derived from *L7-PP2B KO* mice (Fig. 6*C*). Concurrently, we observed an enhanced miniature EPSC amplitude in their PCs recorded from acute slices (Fig. 6*D*,*E*), in line with the observed changes in the size of the clusters. Thus, in contrast to a reduction in the synaptic pool of GluR2 (Fig. 2), the surface and functional GluR2 subunits appear to be increased, which may reflect a compensation to maintain basal activity and homeostasis in PCs (De Zeeuw et al., 2011). So, even with more receptors at the membrane (physiologically detectable) the total level of receptors (surface and internal protein level) may go down, in which the internal pool depletion simply outnumbers the surface receptors. Conjunctively, our data indicate that both the lateral mobility and the total surface expression of GluR2-containing glutamate receptors at PF-PC synapses depend on the phosphatase activity of PP2B, and that this mechanism may be further engaged when basal activity and thereby survival of PCs needs to be warranted.

### Motor learning is partially rescued by enzyme-dead PP2B expression

**Figure 7.**
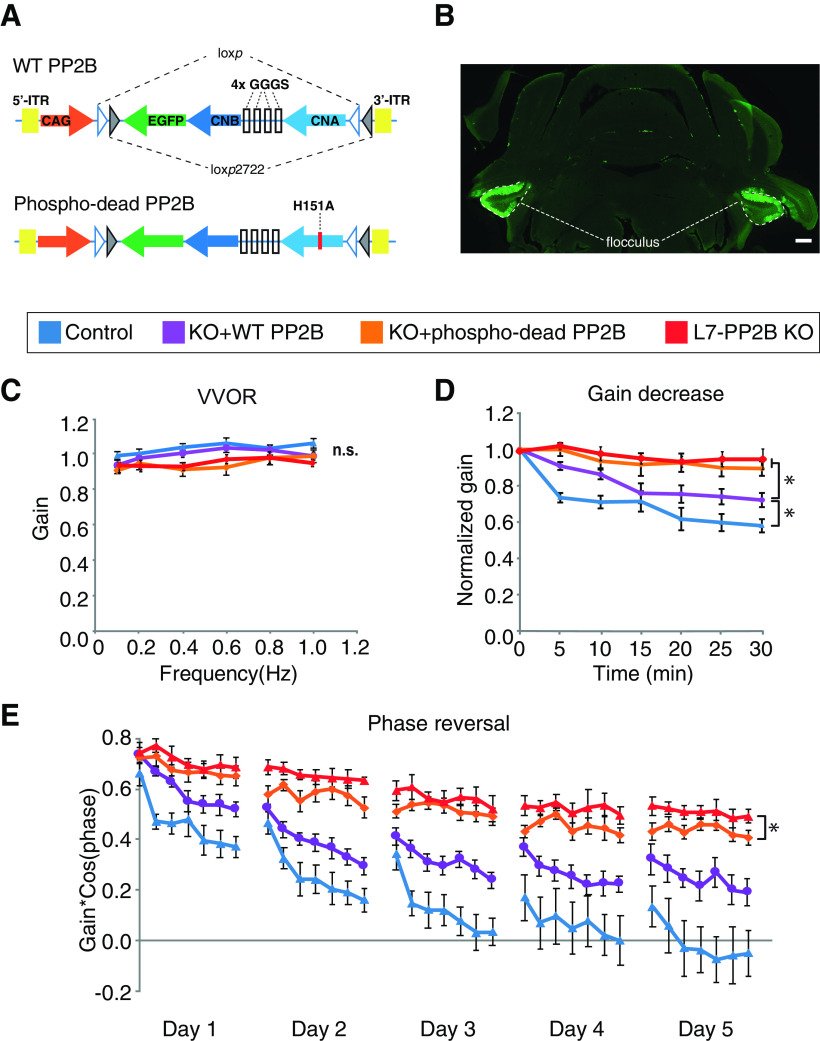
Expression of enzyme-dead PP2B partially rescues motor learning deficits in *L7-PP2B KO*s. ***A***, Schematic of the Cre-dependent WT-PP2B and enzyme-dead PP2B AAVs. The enzyme-dead PP2B is made by introducing a single amino-acid mutation in CNA at H151A. ***B***, Example of bilateral injections of AAV into the flocculus. Text box below represents the experimental groups. Blue represents control (WT littermates injected with CAG-EGFP, *n* = 9 mice). Purple represents WTR (*L7-PP2B KO*s reexpressed with WT-PP2B, *n* = 11 mice). Orange represents PD (*L7-PP2B KO*s injected with enzyme-dead PP2B, *n* = 12 mice). Red represents *L7-PP2B KO* (*n* = 7 mice). ***C***, Motor performance of VOR in light (VVOR). ***D***, Short-term learning paradigm for gain-decrease (ANOVA with Bonferroni correction, *p* < 0.001; PD vs WTR: *p* = 0.015; PD vs control: *p* < 0.001; KO vs control: *p* <0.001; KO vs WTR: *p* = 0.007; KO vs PD *p* > 0.5; control vs WTR: *p* = 0.031) *p < 0.05. ***E***, Long-term learning paradigm following 5 d phase-reversal training. *y* axis indicates the value of gain*cos(phase); the lower this value, the better the learning. Phase-reversal comparison for the last 2 d showed that *L7-PP2B KO* mice injected with PD learned better than the *L7-PP2B KO* mice (unpaired *t* test, *p* = 0.038). Control WT mice injected with GFP and *L7-PP2B KO*s with WT-PP2B learned better than the PD KO group, indicating that both the enzymatic and nonenzymatic functions of PP2B may play a role in VOR learning.

Given the contribution of PP2B to the expression of synaptic proteins and synaptic function, we sought to assess whether reinstating PP2B into the cerebellum can restore motor performance and learning. Thereto, we bilaterally injected AAV containing native PP2B or enzyme-dead H151A mutant PP2B constructs (WTR or PD; Fig. 7*A*) into the flocculi of adult *L7-PP2B KO* mice or in L7-cre mice for control. Such injections resulted in a PC-specific reexpression of PP2B in ∼50% of the cells (Fig. 7*B*). All animals showed a relatively normal eye movement performance in that the VVOR values among the four different groups were not significantly different (Fig. 7*C*). However, during eye movement, learning several differences were observed. The *L7-PP2B KO* mice with reexpression of native PP2B showed a partial, yet significant, improvement in the gain-decrease paradigm (*p* = 0.006; Fig. 7*D*). Furthermore, both types of rescue (i.e., with the WTR or the PD constructs) showed a significant, yet partial, improvement (*p* = 0.00027 and *p* = 0.038, respectively) in the phase-reversal paradigm (Fig. 7*E*). In both cases, the enzymatic impact of PP2B was also evident in that there was a significant difference (*p* = 0.013 and *p* = 0.011, for gain-decrease and phase-reversal, respectively) between the reexpression of the complete PP2B and expression of the enzyme-dead mutant PP2B (Fig. 7*D*,*E*), further complementing the conclusion that enzyme-dead mutant PP2B can only partially rescue the behavioral defects. Together with the phosphatase inhibition experiments (Fig. 1), these data indicate that PP2B facilitates compensatory eye movement learning through both its enzymatic and nonenzymatic (i.e., structural function).

## Discussion

In this study, we explored the functional role of PP2B, one of the most abundant phosphatases in the brain. Specific genetic deletion of PP2B in PCs affects the expression of several PSD proteins at their PF synapses as well as the shape of their postsynaptic densities. IP experiments showed that these PSD proteins likely act as true interactors that bind to PP2B. Moreover, complete deletion of PP2B in PCs causes more severe learning deficits than the inhibition of its phosphatase activity alone. Our data reveal, for the first time, that PP2B plays a structural role in the composition of the PSD, next to its enzymatic role.

### PSD proteins affected by ablation of PP2B

Our quantitative proteomics and subsequent immunoblot experiments demonstrated alterations of the composition of PSD proteins at the PF-PC synapse in *L7-PP2B KO* mice. Interestingly, these included well-studied proteins relevant for PSD structure and function (Homer3, Shank1/2, CaMKII) as well as glutamate receptors or (auxiliary) subunits (mGluR1, GluR2, Grid2, Shisa6). These proteins were found to interact with PP2B, and their levels were reduced after PP2B deletion. This argues for a structural role of PP2B in the maintenance or stability of these proteins in the synapse. In contrast to the changes observed after deleting PP2B from GCs (as studied in the α6-PP2B KO mice), we did not observe any alteration of presynaptic proteins in the PC-specific *L7-PP2B KO* mice. Given that GCs form the source of the major presynaptic input to PCs (i.e., the PFs) (Ito, 2002), this result indicates that the major impact of the genetic ablation of PP2B in the *L7-PP2B KO* mice was indeed restricted to the postsynaptic side.

Many of the PSD proteins that were expressed at a lower level in the *L7-PP2B KO* mice formed large molecular weight complexes with PP2B at PF-PC synapses. This finding is consistent with the observation that many of these PSD proteins are clustered, forming large protein complexes, such as Homer3/Shank1/mGluR3 or mGluR1/Shank2/Grid2 (Uemura et al., 2004). Several of these complexes appear relevant for the control of synaptic plasticity. For example, Homer3, which is the predominant isoform of Homer proteins in PCs (Furuichi et al., 2008), can regulate intracellular calcium by binding to mGluR1 via ER-associated ITPR1 receptors (Tu et al., 1998; Xiao et al., 1998) and thereby control the balance of LTP and LTD induction at PF-PC synapses (Coesmans et al., 2004). Likewise, the presence and/or activity of mGluR1, Shank2, Grid2, and CaMKII is crucial for the balance of LTP and LTD induction at PF-PC synapses in a calcium-dependent fashion (Uemura et al., 2004; Peter et al., 2016). Thus, PP2B in PCs appears to be integrated in larger molecular complexes at their synaptic PF inputs, which are likely to play a role in plasticity and learning.

### Impact of PP2B on structure of PF-PC synapses

When we observed the lower levels of several PSD proteins in PP2B KO mice, the interaction of PP2B with these proteins, as well as the binding of PP2B to PSD proteins in complexes with large molecular weights, we hypothesized that the absence of PP2B may affect the PSD structure. The shape of the PSD of PF-PC synapses indeed turned out to be significantly altered in the *L7-PP2B KO* mice in that their relative thickness (i.e., ratio thickness/length) was significantly reduced. As the average area of the PSDs (thickness × length) in our two-dimensional analysis of the electron microscopic sections remained constant among mutants and controls, one consequence of the relative elongation in shape may be that receptors in the synaptic membrane might move across a more spread out, larger area in the mutants (because of their thinner PSD complex in 2D). This is compatible with what we observed using dSTORM (Dani et al., 2010; Huang et al., 2010). This analysis showed that the density sizes of different PSD proteins were differentially affected through phosphatase-dependent and -independent mechanisms in the *L7-PP2B KO* PCs, implying that the distribution of these proteins is altered.

### Impact of PP2B on mobility of AMPARs at PF-PC synapses

Our single-molecule tracking experiments showed that the movements of surface GluR2-containing AMPARs were significantly affected in a phosphatase-dependent fashion. These data are in line with the fact that lateral mobility of AMPARs is at least partly controlled by site-specific phosphorylation of various PSD proteins (Constals et al., 2015; Penn et al., 2017). Thus, we speculate that the deficits in complexes of PSD proteins in the *L7-PP2B KO* mice contributed to alterations in the structure of their PF-PC synapses as well as in the mobility of their glutamate receptors, together reducing the capacity for cerebellar learning.

### Enzymatic and structural role of PP2B in learning

Cerebellar learning has been postulated to be largely mediated by plasticity at the PF-PC synapse (Marr, 1969; Albus, 1971; Ito, 2002). Possibly, opposing types of plasticity, such as LTP and LTD, may be the dominant forms of plasticity for upbound or downbound modules, dependent on whether the simple spike frequency of PCs needs to increase or decrease during learning, respectively (De Zeeuw, 2021). Inspired by these ideas, many cerebellar studies in mouse mutants have been designed to address the putative relation between motor learning behavior and PF-PC plasticity by genetically manipulating one of the (de)phosphorylating enzymes involved in the induction of synaptic plasticity, such as the protein kinases PKC (De Zeeuw et al., 1998; Xia et al., 2000), cGKI (Feil et al., 2003), and α/βCaMKII (Jörntell and Hansel, 2006; van Woerden et al., 2009), or the protein phosphatases PP2B (Schonewille et al., 2010) and PTPRR (Erkens et al., 2015). Likewise, mutating the phosphorylation sites of AMPARs (e.g., serine or threonine residues) has shed light on the relation between cerebellar learning and synaptic plasticity at the PF-PC synapse (Schonewille et al., 2011; Boele et al., 2018). However, it remained to be determined whether the enzymatic activity of these proteins was the sole factor responsible for mediating cerebellar learning. Here, we show that, unlike the genetic deletion of PP2B in PCs, pharmacological inhibition of the enzymatic function of PP2B was not sufficient to affect baseline motor performance and only partially affected gain-down and phase-reversal learning. Since systemic injection of FK506 was as potent in inhibiting the enzymatic activity of PP2B as the genetic deletion, the limitations of these behavioral effects on cerebellar learning support the possibility that PP2B also exerts a nonenzymatic, potentially structural effect. This hypothesis was further corroborated by the finding that proper cerebellar motor learning in the *L7-PP2B KO* mice was only possible when we reexpressed a fully operational PP2B that exerted not only its phosphatase, but also its nonenzymatic, structural function. These behavioral results highlight that PP2B may not only act as a phosphatase, but also as a structural protein, together facilitating behavioral learning.

### General implications

PP2B has long been considered to function only as a phosphatase. The current study raises the possibility of a novel structural role for PP2B in regulating the PSD protein composition as well as the shape of the postsynaptic density. PP2B is interacting with several PSD proteins, yet the reduction of PSD proteins in the *L7-PP2B KO* mice does not appear to be correlated with a net hyperphosphorylation of most of these proteins. Possibly, PP2B mainly acts as a scaffold for these proteins to sit on. As a consequence, absence of PP2B will directly result in a reduction in the actual levels of these proteins at the synapse and reexpression of PP2B should directly restore this. Indeed, the impaired density of homer3 and mGluR1 of PP2B KO PCs was restored after expressing a phosphatase dead PP2B.

A similar enzymatic independent role in controlling synaptic plasticity has been reported previously for CaMKII (Hojjati et al., 2007; Hell, 2014). Using targeted mouse mutants and pharmacologic inhibition of αCaMKII, Hojjati et al. (2007) demonstrated that the structural presence of αCaMKII protein itself, but not its activation, autophosphorylation, or its ability to phosphorylate synapsin I, is required for normal short-term presynaptic plasticity at hippocampal CA3-CA1 synapses. In contrast to the potential structural function of PP2B in the PSD, the deficits in lateral mobility of AMPARs could not be rescued by phosphatase dead PP2B. As highlighted above, it is quite possible that the lateral mobility of glutamate receptors is solely regulated by the PP2B phosphatase function. Since we were also unable to rescue the density of key PSD proteins, such as Shank1 and Grid2, with the phosphatase-dead PP2B, it is also possible that a compromised PSD by itself might further hamper proper receptor function.

It is intriguing that by reexpressing native PP2B, we were able to rescue all the deficits in *L7-PP2B KO* mice, ranging from PSD protein distribution to cerebellar learning. This argues against the possibility that the genetic deletion of PP2B in the *L7-PP2B KO*s causes developmental deficits in PCs and cerebellar circuits that lead to irreversible functional deficits. Rather, it is likely that PP2B is constantly required for proper synaptic function and motor learning. While expressing PP2B in nearly half of the floccular PCs was sufficient to restore baseline performance and partially rescue motor learning, it is conceivable that restoring PP2B activity in all PCs could rescue the learning deficits for an even larger part.
